# Extragalactic Science with the Orbiting Astronomical Satellite Investigating Stellar Systems (OASIS) Observatory

**DOI:** 10.1007/s11214-023-00948-0

**Published:** 2023-02-02

**Authors:** Susanne Aalto, Cara Battersby, Gordon Chin, Leslie K. Hunt, Dimitra Rigopoulou, Antony A. Stark, Serena Viti, Christopher K. Walker

**Affiliations:** 1 Department of Space, Earth and Environment, Onsala Space Observatory, Chalmers University of Technology, Onsala, Sweden; 2 Department of Physics, University of Connecticut, Storrs, 06269 CT USA; 3 Planetary Systems Laboratory, NASA Goddard Space Flight Center, 8800 Greenbelt Road, Greenbelt, 20771 MD USA; 4 Istituto Nazionale di Astrofisica-Osservatorio Astrofisico di Arcetri, Firenze, Italy; 5 Department of Physics, University of Oxford, Parks Road, Oxford, OX1 3PU UK; 6 Center for Astrophysics | Harvard & Smithsonian, 60 Garden St., Cambridge, MA USA; 7 Leiden Observatory, P.O. Box 9513, Leiden, The Netherlands; 8 Department of Astronomy and Steward Observatory, University of Arizona, Tucson, 85719 AZ USA

**Keywords:** Extragalactic science, THz spectroscopy, Heterodyne spectral resolution, Flight mission concept

## Abstract

The *Orbiting Astronomical Satellite for Investigating Stellar Systems (OASIS)*, a proposed Astrophysics MIDEX-class mission concept, has an innovative 14-meter diameter inflatable primary mirror that will provide the sensitivity to study far-infrared continuum and line emission from galaxies at all redshifts with high spectral resolution heterodyne receivers. *OASIS* will have the sensitivity to follow the water trail from galaxies to the comets that create oceans. It will bring an understanding of the role of water in galaxy evolution and its part of the oxygen budget, by measuring water emission from local to intermediate redshift galaxies, observations that have not been possible from the ground. Observation of the ground-state HD line will accurately measure gas mass in a wide variety of astrophysical objects. Thanks to its exquisite spatial resolution and sensitivity, *OASIS* will, during its one-year baseline mission, detect water in galaxies with unprecedented statistical significance. This paper reviews the extragalactic science achievable and planned with *OASIS*.

## Extragalactic Capability of OASIS

**Fig. 1:**
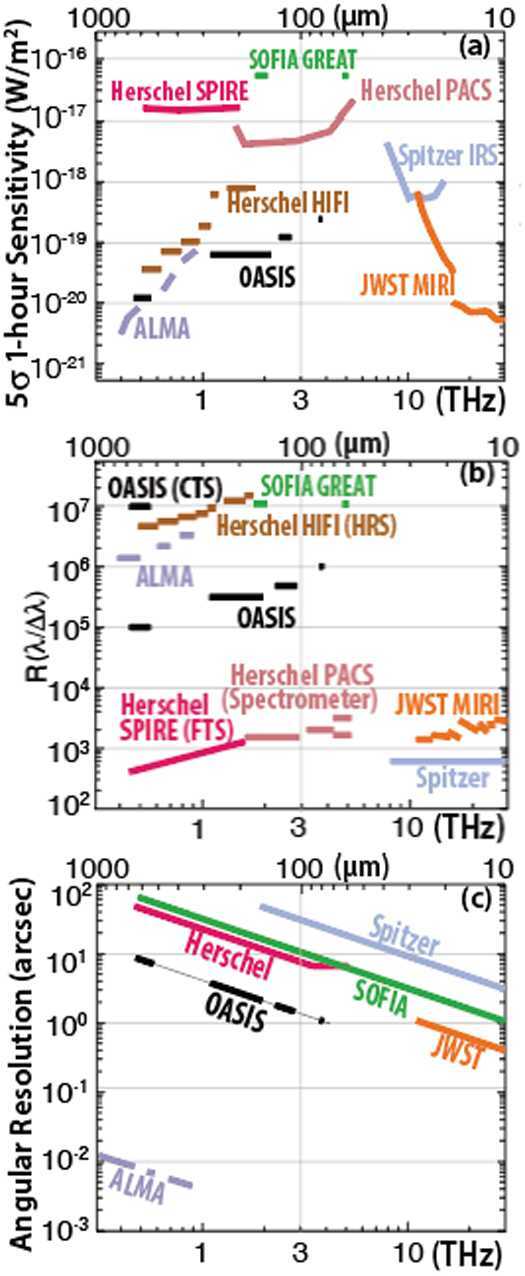
Spectral line sensitivity, resolving power, and angular resolution comparisons of *OASIS* and other far-infrared telescopes. Top: *OASIS* spectral line sensitivity is comparable to that of *JWST* and ALMA, and one or more orders-of-magnitude better than *Herschel* or *SOFIA*, Lower values of sensitivity are better. Middle: *OASIS* spectral resolving power is comparable to the best available with *SOFIA* and ALMA, and (previously) with *Herschel HIFI*, all hetrodyne detectors. Higher values of spectral resolving power are better. Bottom: *OASIS* will have nearly an order-of-magnitude improvement in angular resolution relative to past and present far-infrared telescopes. Lower values of angular resolution are better

*OASIS* is a proposed MIDEX-class mission concept to place, beyond the Earth’s atmosphere, a telescope with a 14-meter diameter collecting area and state-of-the-art cryogenic heterodyne receivers operating at terahertz (THz) frequencies. Its capabilities will bring an unparalleled enhancement to extragalactic science. The 14-meter mirror is a novel inflatable structure design. Its large size enables more than $16\times $ the sensitivity and $4 \times $ the angular resolution of the *Herschel Space Observatory*, the previous comparable space telescope. It complements the capabilities of both the *James Webb Space Telescope* (*JWST*), which operates at shorter wavelengths, and the *Atacama Large Millimeter Array* (*ALMA*), which operates at longer wavelengths. *OASIS’* heterodyne receivers will enable high spectral resolution observations (resolving power $>10^{6}$) of molecular and atomic spectral lines. These frequencies encompass far-infrared transitions of water and its isotopologues, deuterated molecular hydrogen (HD), and spectroscopic lines of other molecular, ionized, and atomic species, at wavelengths from 660 to 63 μm. These wavelengths are obscured by Earth’s atmosphere and cannot be observed from the ground. Comparisons to other far-infrared telescopes are shown in Fig. 1. Details of the design and capabilities of *OASIS* are described in Walker et al. (2021), and some of the material below was presented at the SPIE 2021 Optics + Photonics conference.

**Fig. 2:**
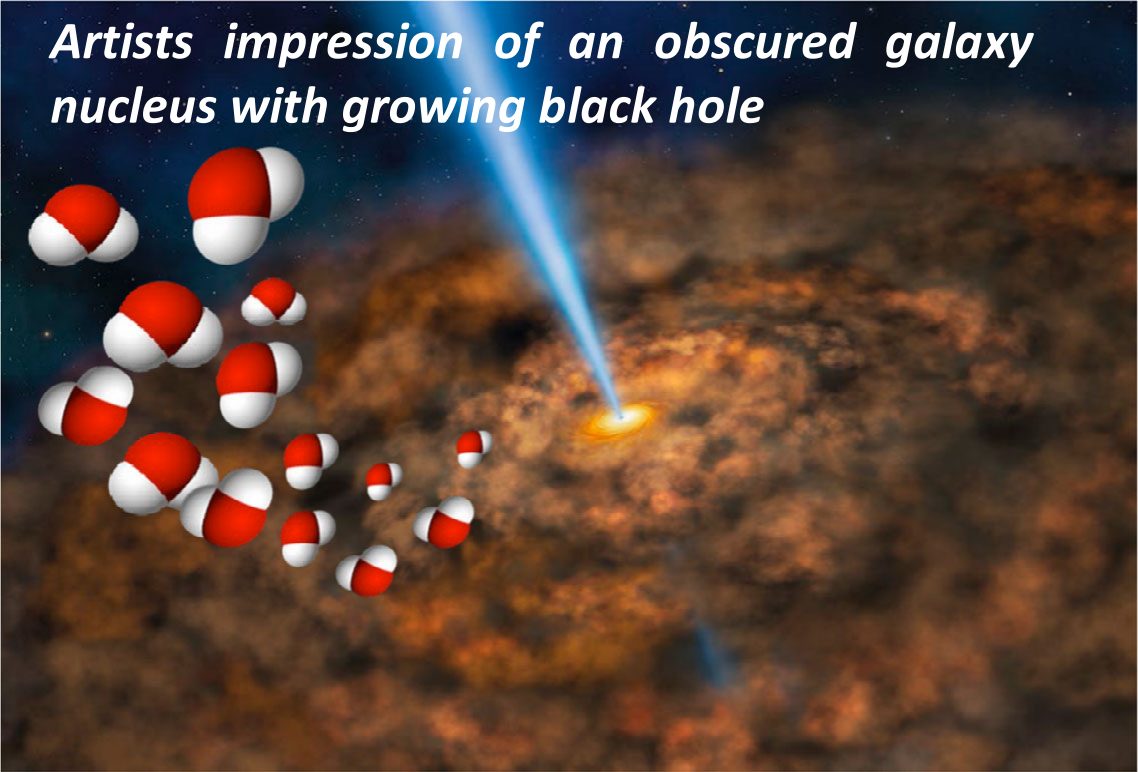
*OASIS* will reveal hidden black holes and stellar nurseries in dusty galaxy nuclei by probing the vast water reservoirs in a complete sample of luminous infrared galaxies (U/LIRGs), including ULIRGs at intermediate redshifts. The water measurements, ions, and especially $\text{H}_{2}^{18}\text{O}$ provide key insights into the most active stages of star formation and growing supermassive black holes. (Image credit: [NASA/SOFIA/Lynette Cook], with added cartoon water molecules)

Star formation and Active Galactic Nuclei (AGN, powered by accreting supermassive black holes, SMBHs, Fig. 2) are fundamental processes that govern the formation, growth, and evolution of galaxies. Much of that development is deeply embedded in interstellar dust, since the bulk of star formation takes place in dense interstellar clouds, and SMBHs go through their most vigorous growth phase while enshrouded in dust and gas (e.g. Noguchi et al. 2010). The regions around obscured AGN span the full range of environments and astrophysical processes that drive the growth of SMBHs, and reflect the connections between the host galaxy and its black hole. Probing inside the inner layers of a galaxy on scales of arcseconds or finer is an observational challenge, since the dust obscures the regions from view at optical and ultraviolet (UV) wavelengths. These regions are therefore a mostly unexplored phase of the growth of black holes and their host galaxies. Studying the hidden, embedded phase of galaxy evolution is key to many unsolved questions about how galaxies co-evolve with their SMBHs, how star formation progresses in extreme dense environments, and how the growth of black holes is regulated. At the longer wavelengths at which *OASIS* will operate, the dust is transparent but glowing, yielding a look deep inside.

In dense interstellar clouds, atoms come together to form molecules. making them valuable tracers of the physical, chemical, and kinematic properties of the obscuring gas. Water forms from the oxygen that is released from exploding massive stars plus hydrogen that originated in the first minutes of the Big Bang. Water can reach extremely high abundances in dense gas, where it serves as a unique signpost of the evolution of the embedded activity (Dutkowska and Kristensen 2022). Water can, however, only form efficiently in the gas phase if temperatures exceed 220 K. In lower temperature environments, water forms on the surface of dust grains. Water that has formed as ice on the surface of dust grains in dense, cold, cores of molecular clouds can be sublimated into water vapour in the gas phase by energetic activity associated with star and planet formation or by growing black holes. Water vapour has a rich spectrum with transitions mainly at submillimeter and far-IR wavelengths, corresponding to terahertz frequencies. Water line emission from high-redshift galaxies can be observed with the *ALMA*; water emission in local and intermediate redshift galaxies is, however, unobservable from the ground, but can be studied with *OASIS* at unprecedented sensitivity and resolution, both spatial and spectral. Water line emission has the useful feature of tracing the far-infrared radiation field, where the obscured activity has its peak energy output.

*OASIS* will follow the path of water in the evolution of galaxies: from the extremely water-rich dusty cores of powerful luminous and ultraluminous infrared galaxies (U/LIRGs) to the detailed study of the embedded accretion processes in nearby, more modest and numerous, star forming galaxies and AGN. U/LIRGs are compact and well-suited for the relatively high resolution of OASIS. With a suite of spectral lines of H_2_O, its isotopomers, and their ionic forms, we can chart the origin and fate of dusty nuclei, and unveil the nature of the buried luminosity sources in a significant sample ($> 100$ objects) of the local U/LIRGs. *OASIS* will also close an important knowledge gap between the luminous distant, high-redshift ($z > 3$) galaxies, and the more local and intermediate (out to $z = 0.8$) redshift U/LIRGs. This epoch is a missing link in understanding the slowing down of the evolutionary processes. *OASIS* also has the unique capacity to detect terahertz fine-structure lines of oxygen, nitrogen and carbon in intermediate redshift galaxies. These lines are key to probing the evolution of nucleosynthesis and astration, and together with the vital water molecule, they complete the picture of energetic dust-enshrouded accretion.

“Metallicity” is a concept used by astronomers to describe enrichment of the interstellar medium (ISM) by the release of processed material from dying stars. Water is an essential part of the oxygen budget of interstellar clouds and *OASIS* will explore how the role of water changes with environment and metallicity. *OASIS*’ exceptional spectral and spatial resolution allow us to follow the path of water through a galaxy: how it transitions from the extended gas in the galaxy disk and into star- and planet-forming clouds. In nearby ($D < 10$ Mpc) galaxies, *OASIS* can resolve structures inside individual molecular clouds, and the uniquely high velocity resolution of the *OASIS* spectrometers enables the separation of features on even smaller scales, the scale of turbulent structures within molecular clouds. A large number of nearby galaxies offer a variety of environments where *OASIS* will study crucial aspects of relations between the properties of interstellar gas and the rate at which that gas forms stars. *OASIS* data will show how black holes, together with their surrounding interstellar medium, evolve in nearby, less powerful, AGN.

With *OASIS* we have the chance to detect water in local low-metallicity galaxies, where water abundances are also expected to be high. Metals are produced by stars, so multiple generations of stars hosted by a galaxy throughout its history result in increasing amounts of “metals” mixed in with the primordial hydrogen and helium. Tracing the path of water into metal poor environments is fundamental to our understanding of galaxy assembly and the conditions of star and planet formation in the early universe. Regions of low metallicity are expected to be more common in the early days of the Universe, but regions of low metallicity still exist today, typically in nearby dwarf galaxies that provide an opportunity to explore conditions akin to those in the early Universe, but at high resolution and sensitivity. The combination of a large collecting area and high spectral resolution lets *OASIS* detect water in these extreme and important environments for the first time.

*OASIS* will also observe deuterated molecular hydrogen, HD, to find hitherto undetected, “dark” molecular gas, which may well be the main molecular phase in metal poor galaxies, but which also shows surprising abundance in regions of more normal, Solar-like metallicity. The dark molecular gas is undetectable with standard molecular tracer molecules (such as carbon monoxide, CO) but can be detected by the faint HD line. The large collecting area of *OASIS* equips it uniquely to map HD emission in nearby galaxies for the first time.

The *OASIS* extragalactic path of water will address fundamental, high-priority science questions that stem from the NASA Astrophysics Roadmap (*Enduring Quests Daring Visions: NASA Astrophysics in the Next Three Decades;* Kouveliotou et al. 2014). One of these, goal 3.3 of the Roadmap, asks, “How did we get here – The history of galaxies.” Dust obscured star formation and black hole growth is fundamental to the evolution of galaxies, for which water is a key diagnostic, in particular in the final, most extreme stages of accretion, where *OASIS* will provide crucial information. Metal-poor dwarf galaxies are also essential ingredients in the history of galaxies, and *OASIS* has the capacity to provide unique information on the role of water in these unusual environments. The history of galaxies is governed by processes that transform diffuse gas into star forming clouds, where *OASIS* will make groundbreaking contributions through its outstanding spatial- and velocity-resolution. In addition, the *OASIS* extragalactic mission addresses major goals under the heading “Understanding the Cosmic Order” in the current Astrophysics Decadal Survey (National Academies of Sciences 2021). In particular, the survey cites the following Science frontier questions: “How do baryons cycle in and out of galaxies, and what do they do while they are there? What controls the mass-energy-chemical cycles within galaxies? How do black holes grow, radiate, and influence their surroundings?” These questions address fundamental queries on the growth and history of galaxies, where *OASIS* will make unique and important contributions. *OASIS* will observe and explore the rich and varied water emission in dusty, luminous as well as metal poor galaxies to get the complete picture of the galactic trail of water from the extended gas in galaxies and into the birth of stars and the growth of black holes.

## Water as a Probe of Hidden Galaxy Nuclei

The peak of galaxy growth (the “Cosmic Noon”) occurred already at redshifts of $z = 1\text{--}3$, but rapid evolution still continues in the local, dusty luminous and ultraluminous infrared galaxies (LIRGs and U/LIRGs with $L_{\mathrm{IR}} \sim L_{\mathrm{bol}}=10^{11}\text{--}10^{12}~L_{\odot }$). They are mainly powered by extreme bursts of star formation and active galactic nuclei. U/LIRGs are often the result of mergers or interactions between gas-rich galaxies where enormous amounts of interstellar matter (ISM) is funneled into the center. U/LIRGs are essential to our understanding of how galaxies evolve and they contribute a fundamental part of the galaxy build-up in the Universe (Sanders and Mirabel 1996; Casey et al. 2014, see right panel of Fig. 7). The infrared emission of U/LIRGs stems from emission absorbed and re-emitted by dust enshrouding the extraordinary starburst and AGN activity. Observing the molecular properties of U/LIRGS is essential for understanding the evolution of present-day galaxies and for studying crucial astrophysical processes in their even more energetic intermediate redshift counterparts. The majority of water vapour lines are at far-IR wavelengths, which can only be detected from space. The *Herschel Space Observatory* detected H_2_O in a number of local luminous galaxies (Falstad et al. 2015; van der Werf et al. 2010; González-Alfonso et al. 2010, 2012; Falstad et al. 2017; Rangwala et al. 2011; Kamenetzky et al. 2012; Weißet al. 2010; van der Tak et al. 2016; Liu et al. 2017) following early studies with the *ISO* satellite (González-Alfonso et al. 2008, 2004, Fig. 3). The H_2_O line emission is strong (Fig. 4), and abundances can become very high ($\text{X}(\text{H}_{2}\text{O})> 10^{-5}$) in the inner regions of U/LIRGs (González-Alfonso et al. 2012; Falstad et al. 2015). The dense gas contains small-sized (1 to 100 nanometer) dust grains on which the formation of water molecules is catalyzed. If the dust temperature increases above $T_{\mathrm{d}} \sim 100~\text{K}$, H_2_O will sublimate from its mantle of ice into the gas phase (van Dishoeck et al. 2013; Sandford and Allamandola 1990). High H_2_O abundances may also stem from gas-phase reactions linked to cosmic ray or X-ray ionization (van Dishoeck et al. 2013). Many U/LIRGs therefore have vast reservoirs of water in their nuclei that hold essential keys to their evolution and the nature of the buried source, and can be explored with *OASIS*.

**Fig. 3:**
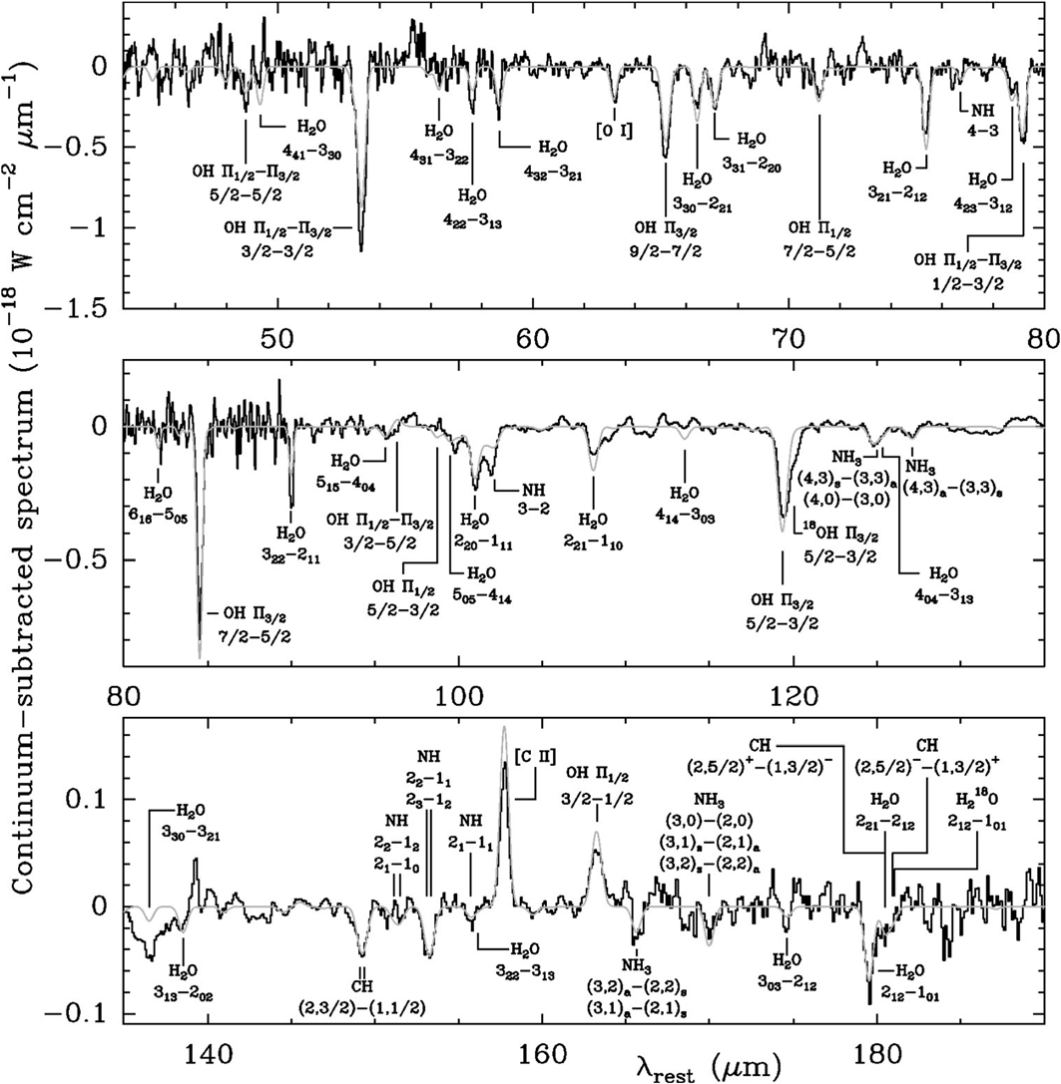
Continuum-subtracted Infrared Space Observatory (*ISO*) spectrum of Arp 220 showing a rich spectrum including prominent H_2_O lines (reprinted from González-Alfonso et al. 2004)

**Fig. 4:**
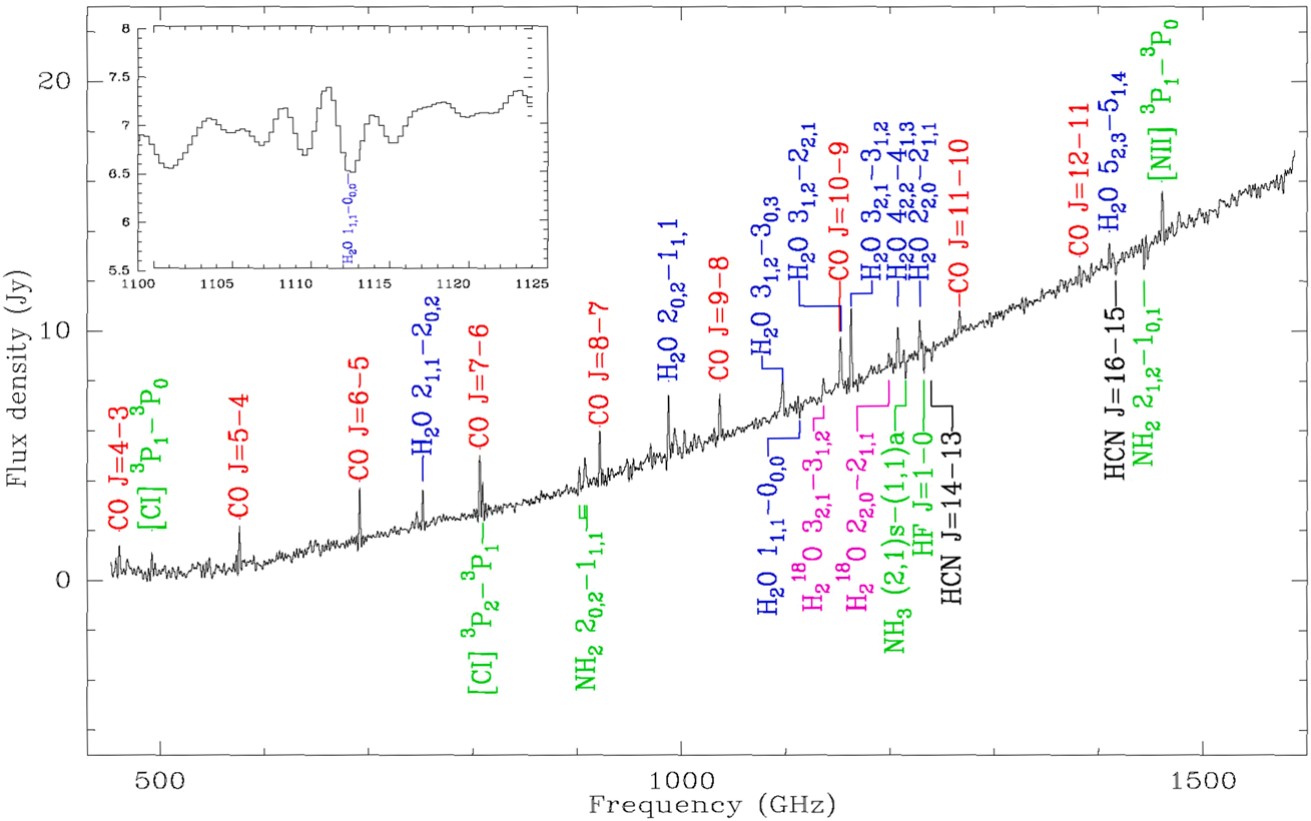
*Herschel* SPIRE spectrum of the water-rich LIRG Zw049.057 revealing luminous, but unresolved, water emission (in blue, reprinted from Falstad et al. 2015). *OASIS* will expand the current sample of U/LIRGs by more than an order of magnitude to fully chart the trail of water in dust embedded nuclei at unprecedented spatial and spectral resolution

**Fig. 5:**
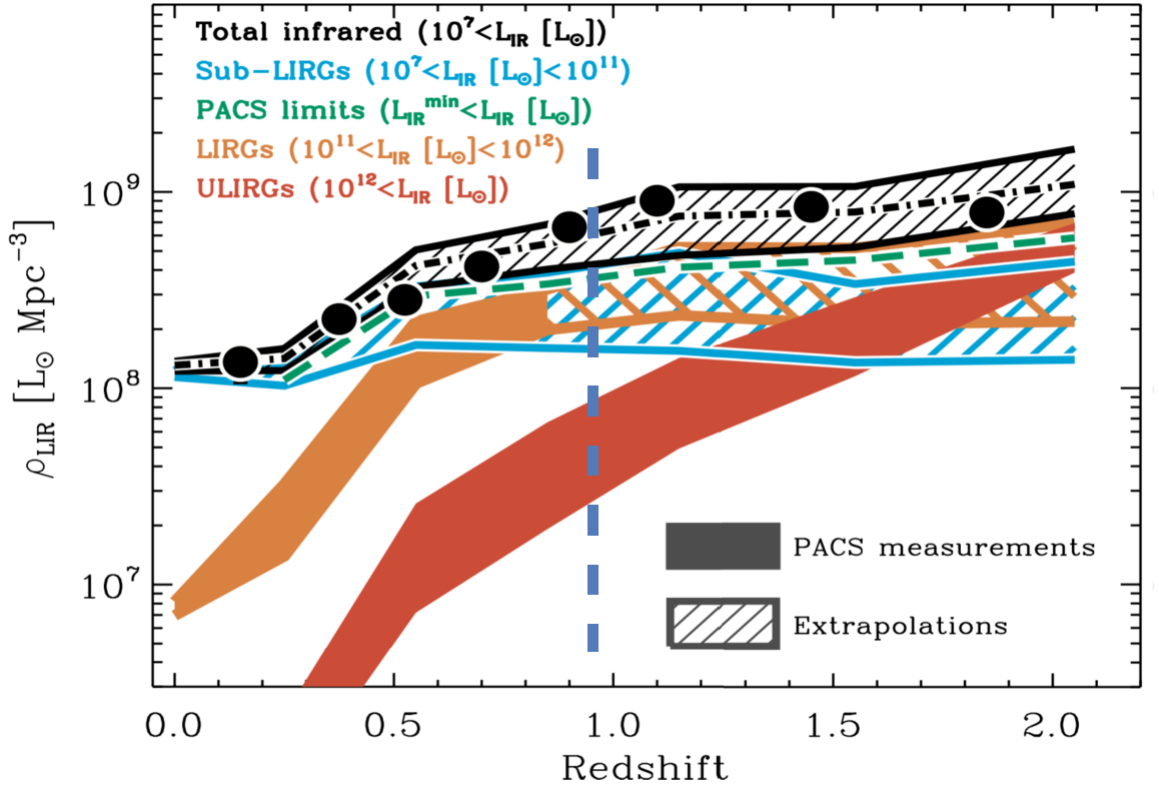
The expected evolution of IR emission density resulting from star formation at redshift $z$. This shows the star Formation Rate Density of the Universe (SFRD, reprinted from Magnelli et al. 2013). The importance of U/LIRGs to the SFRD increases with $z$. *OASIS* will fill in the crucial gap from $z = 0$ to $z \sim 1$ that is inaccessible to ground-based telescopes because of the opacity of water vapor in the Earth’s atmosphere. The range $0 < z < 2$ is crucial because this is where a significant change in the SFRD occurs (cf. Sect. 4)

Furthermore, H_2_O can couple strongly to the radiation field in warm regions with intense far-IR emission (González-Alfonso et al. 2014) where the excitation depends on the radiation density. H_2_O is therefore an excellent tracer of dusty galaxy nuclei, their far-IR radiation fields (cf. Fig. 5), and hidden, embedded luminous sources (González-Alfonso et al. 2014; Falstad et al. 2015; Liu et al. 2017; Yang et al. 2013). Highly excited lines of H_2_O (e.g., at $\lambda = 248.2$, 212.5, and 71.9 μm) have proven efficient in identifying compact obscured nuclei (González-Alfonso et al. 2012, 2014; Falstad et al. 2015) and enshrouded AGN (Liu et al. 2017).

We will compare lines of ortho- and para-water to address the thermal history of the observed water. *OASIS* will simultaneously observe $\text{H}_{3}\text{O}^{+}$, $\text{H}_{2}\text{O}^{+}$ and OH (the main gas-phase precursors of water) leading to a complete account of the chemical network and formation of water (van Dishoeck et al. 2013). The water ions and OH will also provide ionization rates to help determine the physical state of the enshrouded sources.

Resolving the line shape shows whether the dusty, water-rich gas is in- or out-flowing, or if it is the result of disk rotation. It also reveals if there are velocity-dependent differences in H_2_O excitation and abundance. Feedback processes in the form of winds or outflows are common in U/LIRGs (Veilleux et al. 2020), but until now, only tentative evidence of H_2_O in outflows (van der Tak et al. 2016; Falstad et al. 2017) has been found.

So far, only a few U/LIRGs in the local universe have had their H_2_O properties studied in detail, and even fewer were observed at high spectral resolution with the *Herschel* HIFI high resolution spectrometer (van der Tak et al. 2016; Liu et al. 2017). OASIS will expand the current sample of U/LIRGs by an order of magnitude to fully chart the trail of water in dust-embedded nuclei. *OASIS* provides an ideal suite of lower and higher excitation lines, together with simultaneous observations of the continuum, that will allow us to determine H_2_O abundances, the buried IR field and the nature of the embedded luminosity source. U/LIRGs are compact and well-suited for the high resolution of OASIS.

## OASIS Will Trace Star Formation Processes with Water Isotopes

A outstanding question in modern astrophysics is how the stellar initial mass function (IMF) – the mass distribution of newly-formed stars – changes in the dense galactic nuclear environment. The IMF affects our understanding of the nature of the embedded luminosity sources as well as the co-evolution of the host galaxy with its black hole. In normal star formation, low mass stars dominate the total mass in the star formation process (Kroupa et al. 2013), whereas in U/LIRG nuclei and around AGN a disproportionate number of massive stars may be born (e.g. Henkel et al. 2014; Aalto et al. 2019; Toyouchi et al. 2022). ^18^O/^16^O isotopolog-ratios are a key tool for investigating the IMF, since models suggest that an elevated ratio is the result of a “top-heavy” IMF (Maeder 1983; Romano et al. 2017). Initial studies indicate raised ^18^O/^16^O ratios in some U/LIRGs (González-Alfonso et al. 2012; Falstad et al. 2015, 2017). The advantage of using the water isotopes is that they target the most embedded regions, which are the hardest to reach with other methods, and are also the locations of the most active evolution. The large improvements in sensitivity and resolution with *OASIS* over Herschel will allow us to carry out systematic studies of water and hydroxyl isotopologs in local U/LIRGs and also in nearby galaxy nuclei harbouring growing supermassive black holes or bursts of star formation (cf. Sect. 7). Our main aims are to search for signatures that the IMF is changing with environment and to follow the evolution of star formation in compact and dust obscured environments.

## OASIS Will Follow the Path of Water and the Evolution of C, N and O out to Intermediate Redshift U/LIRGs

The high sensitivity of *OASIS* enables observations of H_2_O in luminous galaxies at low and intermediate redshifts, from zero out to $z \sim 0.8$. This is an important part of the evolutionary history of the universe, including times when the Star Formation Rate Density (SFRD) and gas fractions (Braun et al. 2011) were higher than they are now. This is an important link to the Cosmic Noon ($z = 1\text{--}3$) where the SFRD reached its peak (Magnelli et al. 2013; Bouwens et al. 2009), and where galaxies with U/LIRG-like luminosities began to dominate the SFRD (Hopkins and Beacom 2006) (see Fig. 5). ULIRGs will give clues to the drivers behind the dramatic rise in SFRD at $z > 1$.

*OASIS* is the only facility that has the wavelength coverage and sensitivity required to access fundamental water lines. (e.g. o-H_2_O 5_23_-5_14_, $\lambda _{\mathrm{rest}}=212.5$ μm; p-H_2_O 3_13_-2_02_ 138.5 μm; and o-H_2_O 4_23_-3_12_ 78.7 μm) in intermediate redshift galaxies. With higher gas fractions and star-forming activity, we expect the water content and luminosity to be higher than in local U/LIRGs. It is unknown, however, in what way the transitional stage of the intermediate redshift galaxies affect their properties in comparison to the local and high-redshift ($z> 3$) Universe, where water emission can be observed from the ground and is found with ALMA (Combes et al. 2012; Omont et al. 2013; Jarugula et al. 2019; Yang et al. 2020, cf. Fig. 7, left panel). Evidence suggesting that the U/LIRG populations in the local and intermediate redshift Universe have disparate properties (Rigopoulou et al. 2014) raises questions about how the fraction of extremely obscured nuclei vary between local and intermediate redshift U/LIRGs and what the impact of water-richness, interaction state, and metallicity have on the efficiency of star formation and on the growth of the black holes.

The large collecting area of *OASIS* allows for complementary and ground-breaking studies of luminous fine-structure lines of oxygen, carbon and nitrogen lines in *unexplored* intermediate redshift galaxies. Most of the elements we see around us today – carbon, nitrogen, oxygen – were formed in massive stars and released into the ISM at the end of the stellar life cycle. The abundance of metals in a galaxy is a key signature of the galaxy’s past history. Gas-phase metallicities represent a direct measure of gas enrichment due to stellar nucleosynthesis and subsequent dispersion of metals in the ISM through stellar winds, supernovae, gas inflow/outflow, and the merger history of a galaxy. Through the determination of metallicities we are able to place constraints on the star formation and intergalactic accretion histories of galaxies, and consequently to constrain models of galaxy formation and evolution. The standard methods to derive gas-phase metallicities are based on UV and optical transitions and are, therefore, susceptible to error due to dust extinction. Such methods are not suitable for determining the metallicities of dust-obscured galaxies such as U/LIRGs. Since a large part of the star-forming activity in the Universe occurs in dust-obscured environments, extinction-free metallicity diagnostics are crucial. Far-infrared fine structure lines provide an alternative way of determining metallicities, avoiding the problem of extinction and the uncertain extinction corrections that plague UV/optical measurements. Lines originating in H II regions probe the ionised component of the ISM and are better suited as tracers of gas phase metallicities as they reflect the metallicity of the gas out of which stars are being formed.

The most sensitive fine structure line ratios to measure metallicities are the [O III] 52 μm, 88 μm and [N III] 57 μm lines (Pereira-Santaella et al. 2017, cf. Fig. 7, right panel), and the [OIII] 88 μm/[NII] 122 μm ratio (Rigopoulou et al. 2018). Far-infrared fine structure lines have been observed in local galaxies with ISO and *Herschel* (Díaz-Santos et al. 2013; Farrah et al. 2013; Rigopoulou et al. 2014; Cormier et al. 2015). With *OASIS* we will measure fine structure lines in intermediate redshift galaxies probing how the star formation activity slowly decreased to reach the levels we see today. For more distant galaxies ($z > 2$) many FIR fine structure transitions are accessible with ALMA (De Breuck et al. 2019), but the FIR fine structure lines are uniquely accessible with *OASIS* in the key epoch at intermediate redshift. Furthermore, we will use the fine structure [C II] 158 μm line as a star formation rate indicator (De Looze et al. 2014; Magdis et al. 2014; Díaz-Santos et al. 2017). The [C II] line may suffer from issues in tracing star formation in the innermost regions of ULIRGs (Dwek and Arendt 2020), but it is still a key probe of the global dusty star formation in luminous galaxies and is essential in determining the SFRD.

## OASIS Will Explore Water in Low-Metallicity Systems

The first galaxies in the Universe formed from a metal-free, pristine interstellar medium, but after the first episode of star formation, they were rapidly enriched with metals from the explosions of the first supernovae (SNe, Wise et al. 2012) These early SNe produced oxygen whose abundance dominated other metals at early times, as shown in Fig. 8. The dominance of oxygen means that young galaxies in the early Universe are expected to be rich in water. They were also metal poor, and their chemical properties cannot, however, be easily studied; chemically, they resemble nearby low-metallicity dwarf galaxies. Dwarfs are the most abundant galaxy type at any cosmic epoch, and are considered to be the “building blocks” of more massive galaxies (Bullock et al. 2000); in the standard hierarchical paradigm, large galaxies grow by accreting smaller ones. From the standpoint of chemical evolution, dwarf galaxies in the Local Universe may be considered good proxies for studying enrichment in the Early Universe.

H_2_O, because of its expected high abundance at low metallicity, should be an important probe of galaxy evolution processes, but so far little, if anything, is known about H_2_O in the dwarf-galaxy regime, because water is not observable from the ground in nearby galaxies and *Herschel* lacked the sensitivity needed to detect them. Interestingly, chemical models predict that in galaxies like the Magellanic Clouds (the two closest dwarf galaxies) the H_2_O abundance is comparable to that of CO. For galaxies in the early Universe, H_2_O is expected to exceed CO (Acharyya and Herbst 2018), making H_2_O a fundamental tracer of physical conditions in the star-forming regions of metal-poor galaxies. Using the exceptional capacity of *OASIS* we will, for the first time, detect this vital molecule in nearby dwarf galaxies, enabling an unprecedented view of dwarf galaxy evolution as a window into the early Universe.

## OASIS Will Use HD to Probe CO-Dark Gas

Carbon monoxide, CO, is often used as a tracer of the molecular mass in galaxies. The H_2_ mass is taken to be linearly proportional to the CO intensity via the so-called X_CO_-factor (Dickman et al. 1986; Downes and Solomon 1998; Bolatto et al. 2013). This is, however, only an approximation. In actuality, extensive observational and theoretical work has shown that a CO-faint phase is possible for molecular material if the average metallicity is low (Cormier et al. 2012) or if strong Far-UV fields are present (Madden et al. 1997; Bell et al. 2006). Models have also indicated that high cosmic ray ionization rates, expected for example in starburst or AGN-dominated galaxies, can destroy CO but not H_2_. This implies that even dense, solar-metallicity regions may host large H_2_ reservoirs which are nevertheless devoid of CO.

Various tracers of this CO-dark gas have been proposed, for example atomic carbon [C I] (Smith et al. 2014a; Bisbas et al. 2015). However, its detection would be restricted to relatively low density, high temperature regimes. Most of the CO-alternatives proposed suffer from either being too low in abundance or, most importantly, not truly able to trace the extent of the H_2_ molecule and hence the molecular mass of a galaxy. An obvious contestant to overcome this limitation is HD, the heavy isotope of molecular hydrogen. The molecular spectrum of HD is entirely different than H_2_, because HD has an electric dipole moment whereas H_2_ has none. The $J=1\text{--}0$ transition of HD is at $\lambda 112~\upmu \text{m}$, and unlike H_2_ it is excited into emission in low-temperature molecular gas. HD is a good tracer of dense molecular gas because at high extinctions HD is well self-shielded and the distribution of HD is expected to closely follow that of H_2_. This has been shown by NASA’s Far Ultraviolet Spectroscopic Explorer (FUSE) mission that detected HD in absorption in the Large Magellanic Cloud (André et al. 2004). Chemical models of dense molecular clouds also show that, above two magnitudes, HD may be a hundred times more abundant than CO (Fig. 9).

Detecting HD has been a challenge so far because the transitions of HD that are excited in the colder phases of the ISM emit at wavelengths where the dust continuum is bright and the Earth’s atmospheric opacity is high. *OASIS* with its large collecting area and high velocity resolution opens new avenues for direct detection using lines of HD that can only be observed from space.

## Water Tracing Feeding and Feedback in Nearby Starburst Galaxies and AGN

**Fig. 6:**
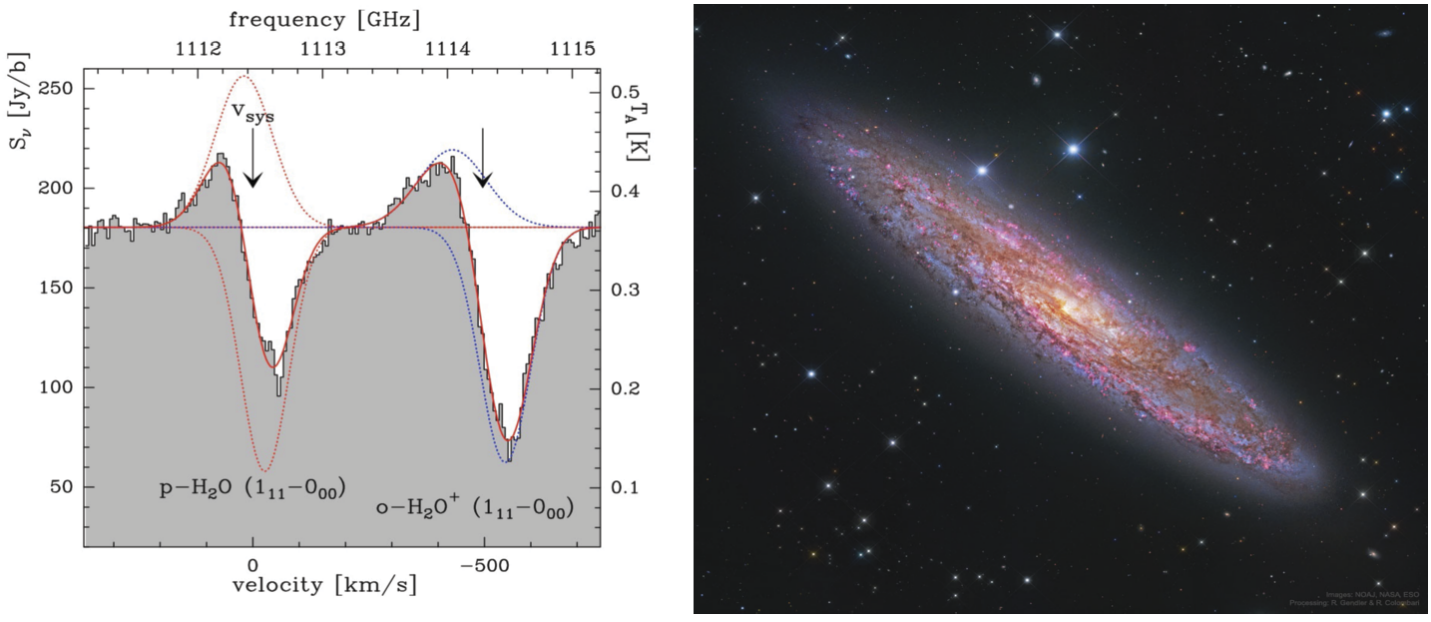
*OASIS* will follow the path of water from diffuse gas to star formation by studying H_2_O, its isotopes and ions at high spatial resolution in nearby galaxy disks and its isotopes. The observations will address crucial aspects of relations between the properties of interstellar gas, the rate at which that gas forms stars, the role of H_2_O in the oxygen budget, and how water ends up in star- and planetary systems. (Left) Prominent H_2_O emission and absorption detected by *Herschel*, (reprinted from van der Tak et al. 2016) in the nearby star-forming galaxy NGC253 (Right)

Because they are relatively rare in the local Universe, U/LIRGs and LIRGs are mostly at distances $> 60$ Mpc. Nearby galaxies ($D < 15$ Mpc) host less extreme central regions than the U/LIRGs, but instead offer an opportunity to follow the trail of water in varying galactic environments at unprecedented spatial resolution (cf. Figs. 6 and 10). Lower luminosity starbursts are more numerous than U/LIRGs and constitute a larger fraction of the local SFRD. Studying them is essential for our understanding of normal, disk-dominated star formation. The strength of *OASIS* is its exceptional collecting area and spectral resolution, which makes it an ideal instrument to study the cycle of water, its ions, and isotopic variants in nearby starburst galaxies and AGN. For the most nearby starburst galaxies, *OASIS* offers spatial resolutions on scales smaller than Giant Molecular Clouds (GMCs). The high velocity resolution of *OASIS* means that we can also identify and study structures on even smaller scales.

**Fig. 7:**
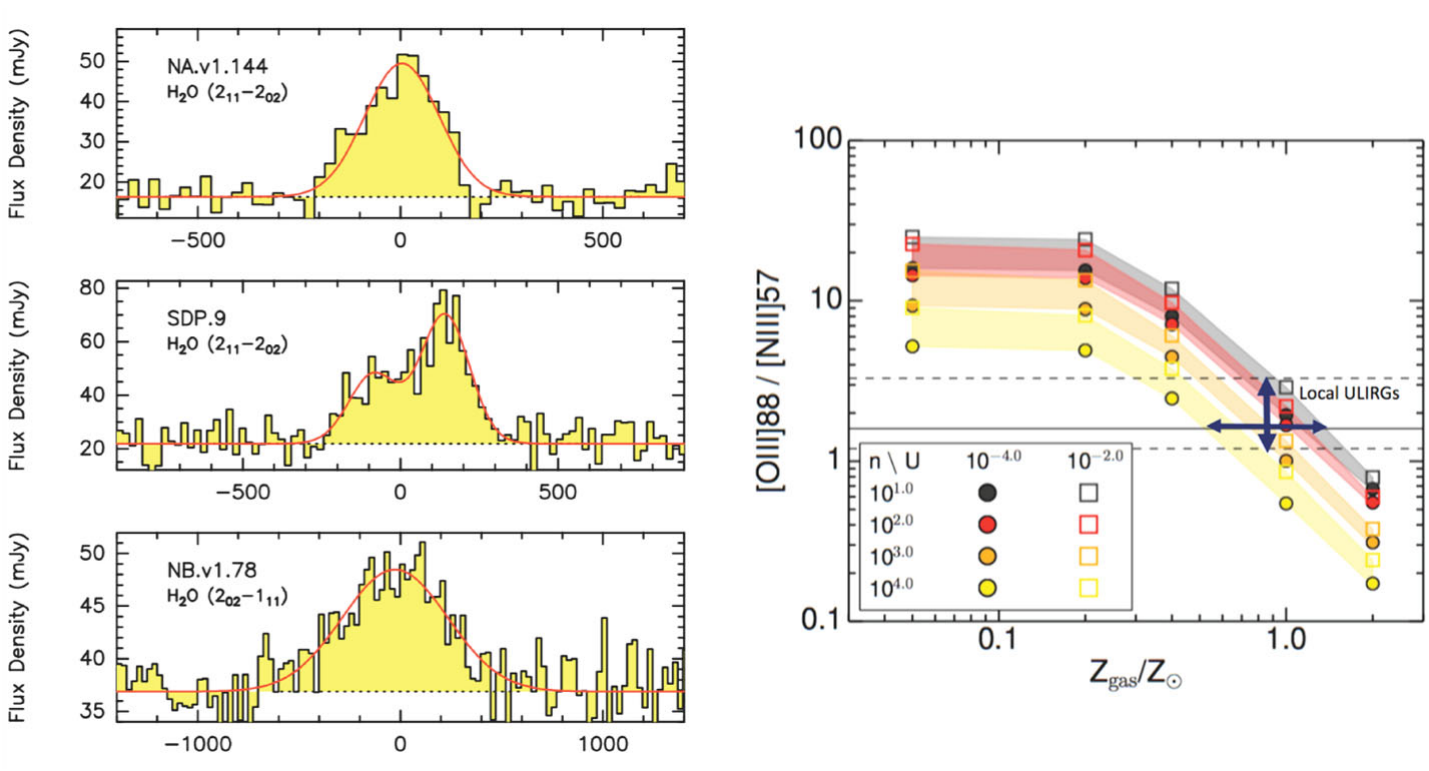
Left: Spectra from IRAM PdBI showing intense water emission in galaxies at redshift $z > 3$ (reprinted from Omont et al. 2013). H_2_O lines are among the strongest molecular lines in high-z U/LIRGs. Right: Metallicity estimates for local U/LIRGs (reprinted from Pereira-Santaella et al. 2017). With *OASIS* we will have the ability to measure water and fine structure lines of O, N and C in a significant sample ($\sim 30\text{--}100$) of intermediate redshift galaxies probing the most recent 5 billion years of the Universe during which the star formation activity slowly decreased to reach the levels seen today

**Fig. 8:**
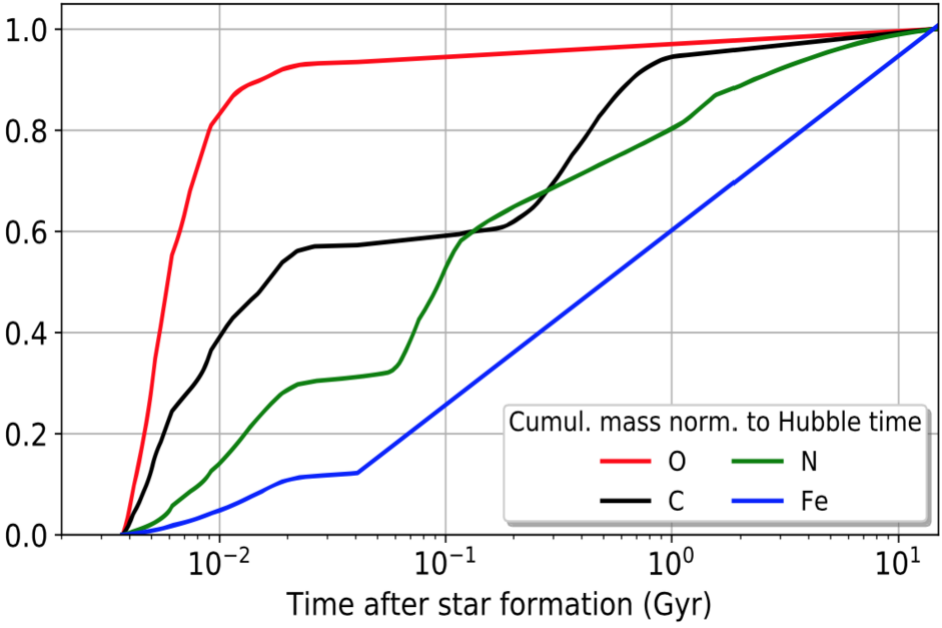
Un-enriched, pristine environments were omnipresent in the early universe. Studying similar regions in the local universe gives key insights into how the first stars were born. OASIS will detect H_2_O for the first time in local metal poor galaxies to reveal its role in early star formation

**Fig. 9:**
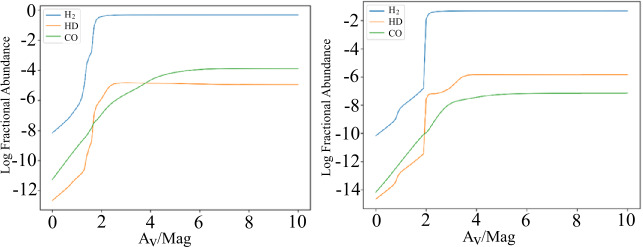
Theoretical predictions of the relative abundance of HD (by S. Viti) with respect to CO and other species as a function of optical depth for molecular gas at a volume density of $10^{4}~\text{cm}^{-3}$, metallicities of 1.0 (left) and 0.1 (right) times the solar system value, and a radiation field 100 times stronger than the Galactic interstellar field. From the figures one can see that between $\sim 2$ and 4 magnitudes of extinction, even for solar metallicity, X(HD) can be > than X(CO). *OASIS*, with its large collecting area and high velocity resolution opens new avenues for direct detection of molecular material using lines of HD that can only be observed from space

**Fig. 10:**
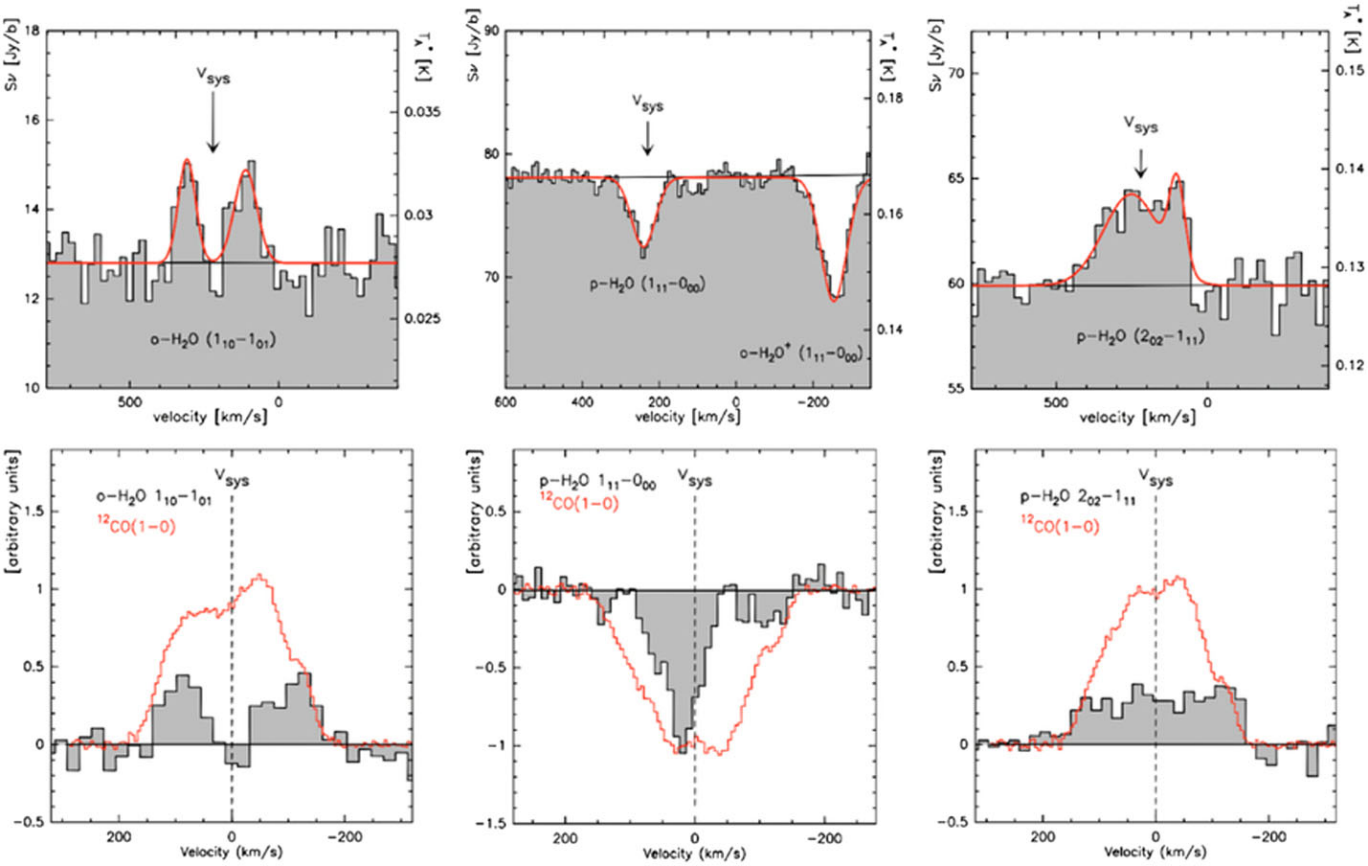
*Herschel* HIFI spectroscopy of low-level water transitions in the nearby starburst galaxy M82 (reprinted from Weißet al. 2010). The top panels show lines of ortho- and para-H_2_O as well as ortho-$\text{H}_{2}\text{O}^{+}$. The lower panels show comparisons with CO profiles (red) at matching resolution. These varying line profiles illustrate the requirement for sufficient spectral resolution. *OASIS* will improve the spatial resolution of existing extragalactic water observations by a factor of ten and will follow the trail of water in a large number of nearby galaxies with varying properties. We will have a complete map of how large- and small-scale processes affect the trail of water in galaxies and the necessary velocity and spatial resolution to disentangle the contributions of the various ISM structures along the line of sight

We will study water in dense star-forming clouds and their connections to the surrounding diffuse molecular gas. This provides essential information on the star formation processes and the delivery of water into dense molecular cloud cores and ultimately into protoplanetary systems. Water is ubiquitous also in the diffuse molecular medium (Weißet al. 2010) and by combining H_2_O lines with OH and water ions we can trace how the water and oxygen chemistry, and excitation, change with environment. Star-forming clouds and inter-cloud gas are often organized into filamentary structures on scales ranging from smaller than 0.1 pc to $>100$ pc (Smith et al. 2014a,b). Filaments may connect small- and large-scale aspects of the star forming process and also link to galaxy-scale dynamical processes such as spiral arms and central bars. Dynamical processes within the star-forming regions occur on parsec and smaller scales – to spectrally resolve them requires velocity resolutions better than $\sim1~\text{km}\,\text{s}^{-1}$, and is uniquely achievable with *OASIS*.

Water excitation and abundance, together with water ions, are excellent tools for probing the evolution of the torus around an AGN. *Herschel* found water in nearby AGN (e.g. Spinoglio et al. 2012; Sandqvist et al. 2021) and *OASIS* will resolve the water emission and accurately determine excitation, water abundances, and kinematics. With *OASIS* we will fully chart the impact of the AGN on the formation and destruction of water in the obscuring torus and how this relates to the AGN luminosity and evolutionary stage.

## Baseline and Threshold Observations

In its 1-year Baseline Science Mission, *OASIS* will observe Solar System objects and protoplanetary objects in addition to the extragalactic program described here. The threshold observational program will observe the cores of at least 100 U/LIRGs at redshifts from $z = 0\text{--}3$ and in a statistically significant sample of local galaxies. Details of how *OASIS* science objectives are scheduled and accomplished during the course of its Baseline Science Mission is presented in the introductory article in this volume.

## Summary

The large inflatable primary mirror of *OASIS* will provide unprecedented collecting area and spatial resolution for heterodyne Terahertz detectors operating beyond the Earth’s noisy, humid atmosphere. *OASIS* will revolutionize our understanding of water in the universe, from the interstellar medium of galaxies to the comets that create oceans. *ALMA* has shown the significance of water in the early universe at high redshift, where the spectral lines are shifted away from mid-infrared atmospheric blockage; *OASIS* will show water’s significance in local and intermediate redshift galaxies in the evolved universe, clarifying the role of water in the present day and in the cores of active galactic nuclei.
